# Molecular Subtyping of *Bacillus anthracis* and the 2001 Bioterrorism-Associated Anthrax Outbreak, United States

**DOI:** 10.3201/eid0810.020394

**Published:** 2002-10

**Authors:** Alex R. Hoffmaster, Collette C. Fitzgerald, Efrain Ribot, Leonard W. Mayer, Tanja Popovic

**Affiliations:** * Centers for Disease Control and Prevention, Atlanta, Georgia, USA

**Keywords:** B. anthracis, subtyping, multiple-locus variable-number tandem repeat analysis (MLVA), pagA

## Abstract

Molecular subtyping of *Bacillus anthracis* played an important role in differentiating and identifying anthrax strains during the 2001 bioterrorism-associated outbreak. Because *B. anthracis* has a low level of genetic variability, only a few subtyping methods, with varying reliability, exist. We initially used multiple-locus variable-number tandem repeat analysis (MLVA) to subtype 135 *B. anthracis* isolates associated with the outbreak. All isolates were determined to be of genotype 62, the same as the Ames strain used in laboratories. We sequenced the protective antigen gene (*pagA*) from 42 representative outbreak isolates and determined they all had a *pagA* sequence indistinguishable from the Ames strain (PA genotype I). MLVA and *pagA* sequencing were also used on DNA from clinical specimens, making subtyping *B. anthracis* possible without an isolate. Use of high-resolution molecular subtyping determined that all outbreak isolates were indistinguishable by the methods used and probably originated from a single source. In addition, subtyping rapidly identified laboratory contaminants and non-outbreak–related isolates.

The recent bioterrorism-associated anthrax outbreak demonstrated the need for rapid molecular subtyping of *Bacillus anthracis* isolates. Numerous methods, including multiple-locus enzyme electrophoresis (MEE) and multiple-locus sequence typing (MLST), have shown the lack of genetic diversity of *B. anthracis* (1–4, unpub. data). Despite this low diversity, methods have been developed that can detect differences between *B. anthracis* isolates. Amplified fragment length polymorphism (AFLP) analysis has been used to detect differences between *B. anthracis* isolates and to examine phylogenetic relationships between *B. anthracis* and its close relatives, *B. cereus* and *B. thuringiensis* (4,5). Keim et al. (6) reported on multiple-locus variable-number tandem repeat analysis (MLVA) for subtyping *B. anthracis*, which unlike AFLP is designed to subtype *B. anthracis* specifically and cannot be used to address phylogenetic relationships between *Bacillus* species. MLVA determines the copy number of variable-number tandem repeats (VNTR) at eight genetic loci (six chromosomal and one on each of the two plasmids). Recently, MLVA has been used to differentiate 426 *B. anthracis* isolates into 89 distinct genotypes and to study the ecology of anthrax (6,7). MLVA is relatively simple, has excellent reproducibility, can subtype multiple strains on a single gel, and gives results in <8 hours.

Protective antigen (PA) is one of the three anthrax toxin proteins and is key to developing immunity to anthrax. Sequencing the gene that encodes PA (*pagA*) has been used to subtype 26 diverse *B. anthracis* isolates into six PA genotypes (8). Although sequencing of *pagA* results in limited numbers of subtypes, it does have the added benefit of determining if the *pagA* gene has been altered or engineered.

During the 2001 bioterrorism-associated anthrax outbreak, we used MLVA to subtype isolates from patients, the environment, and powders. Subtyping of *B. anthracis* allowed anthrax cases to be linked to environmental specimens and powders and provided information about potential sources. Sequencing of *pagA* was also performed on a subset of these *B. anthracis* isolates, and we confirmed that the *pagA* sequence was not altered. In addition, we used these methods on DNA extracted from select clinical specimens to detect and subtype *B. anthracis* directly from clinical specimens. During the outbreak, laboratories throughout the United States and around the world received an increased number of specimens to be tested for *B. anthracis*. With such large numbers, occasional contamination or detection of non-outbreak strains was inevitable, and molecular subtyping was used to clarify these situations on several occasions. Overall, the recent anthrax outbreak has dramatically illustrated the importance of rapid molecular subtyping during a bioterrorism event.

## Materials and Methods

During the 2001 anthrax outbreak investigation, 135 *B. anthracis* isolates were subtyped. The identity of all strains was confirmed with standard microbiologic procedures and the Laboratory Response Network (LRN) testing algorithm (9,10). Isolates were obtained from patients with laboratory-confirmed anthrax (n=10), powders (n=4), and environmental specimens (n=121). For comparison purposes, five *B. anthracis* isolates originating from New England in the 1960s and 1970s, the Ames strain, and the Pasteur strain were included.

DNA extractions of 28 clinical specimens from six patients with confirmed inhalational anthrax were used for molecular subtyping. These specimens included blood, pleural fluid, blood cultures, serum, cerebrospinal fluid (CSF), lung tissue, and lymph node tissue.

DNA from all strains was prepared with a heat lysis method. Isolates were streaked onto trypticase soy agar containing 5% sheep blood (Becton Dickinson Microbiology Systems, Cockeysville, MD) and incubated overnight at 37°C. A single colony was transferred and dispersed into 0.22-µm centrifugal filter units (Millipore, Bedford, MA) containing 200 µL 10 mM Tris-HCl (pH 8.0). The suspension was heated at 95°C for 20 min and cooled to room temperature. The filter units were then centrifuged in a microfuge at 6,000 x *g* for 2 min and the filter discarded. The resulting lysate was stored at –20°C until use. DNA from clinical specimens was extracted with a Qiagen DNA Mini Kit per manufacturer’s instructions (Qiagen Inc., Valencia, CA).

MLVA typing was done as described by Keim et al. (6). Briefly, the eight loci were amplified in four reactions: reaction 1 (*vrrB*_1_, CG3, and *vrrA*), reaction 2 (*vrrB*_2_, pXO1-aat, and pXO2-at), reaction 3 (*vrrC*_1_), and reaction 4 (*vrrC*_2_). In some instances CG3 was removed from reaction 1 and amplified as a 5th reaction because of weak amplification. Each amplicon was labeled with one of three different dyes. Products were separated by polyacrylamide gel electrophoresis under denaturing conditions on an ABI 377 automated DNA sequencer (Applied Biosystems, Foster City, CA), and allele sizes were determined using ABI Genescan software (Applied Biosystems).

The amplification and sequencing of *pagA* were performed on 42 *B. anthracis* isolates and 22 clinical specimens as described by Price et al. (8), with the following modifications. Initially, synthetic oligonucleotide polymerase chain reaction (PCR) primers PA-1F and PA-1R and PA-2F and PA-2R (Table 1) were used to amplify two overlapping fragments (1,119 bp and 1,449 bp, respectively) together totaling 2,531 bp and containing the *pagA* open reading frame (ORF) (8). Because of inconsistent amplification with PA-2F and PA-2R and to generate a single template for sequencing, PCR amplification was performed using primers 1566F and 4205R. In some instances, possibly from the method of DNA purification, 1566F and 4205R did not amplify sufficiently and thus nested PCR was performed using 1575F and 4191R. The primers used in this study were a combination of both published primers (8) and primers designed from the published DNA sequence of the virulence plasmid pXO1 (GenBank accession no. AF065404) (Table 1). The *pagA* sequencing template was amplified by PCR using the Expand High Fidelity PCR system (Roche, Mannheim, Germany). Fifty-microliter PCR mixtures contained 10 mM Tris-HCL (pH 8.3), 50 mM KCl, 4.0 mM MgCl_2_, 0.4 mM of each forward and reverse primer, 100 µM of each deoxynucleotide, 2.0 U of *Taq* DNA polymerase (Roche), and 2 µL of bacterial lysate. Reactions were heated at 94°C for 5 min and then cycled 35 times at 94°C for 30 s, 51°C for 30 s and 72°C for 1.5 min, with a final extension of 72°C for 5 min. PCR amplicons were purified using QIAquick PCR purification kit (Qiagen, Inc.) and the resulting purified amplicons were used in the subsequent sequencing reactions.

**Table 1:** Primers used for amplification and sequencing of the *pagA* gene

Primer	Sequence (5´ – 3´)
PA1575F^a^	CGA ACT GAT ACA CGT ATT TTA G
PA4191R^a^	AGG ATT ATG ATG ATT TAG ATT ACT
PA1566F^a^	TTT ATC CGA ACT GAT ACA CGT ATT
PA4205R^a^	ACA AAC AAT CTC AAA GGA TTA TGA
PA-1F^a^	ATA TTT ATA AAA GTT CTG TTT AAA AAG CC
PA-1R^a^	TAA ATC CTG CAG ATA CAC TCC CAC
PA-2F^a^	ATA AGT AAA AAT ACT TCT ACA AGT AGG ACA C
PA-2R^a^	GAT TTA GAT TAC TGT TTA AAA CAT ACT CTC C
PA-3	TCA TGT AAC AAT GTG GGT AGA TGA C
PA-4	CTC TAT GAG CCT CCT TAA CTA CTG AC
PA-5F	ATC CTA GTG ATC CAT TAG AAA CGA C
PA-5R	CTT CTC TAT GAG CCT CCT TAA CTA CTG
PA-5F_nest_	AGT GAT CCA TTA GAA ACG AC
PA-5R_nest_	TAA CTA CTG ACT CAT CCG C
PA-U2121	TAC ATT TGC TAC TTC CGC TGA TAA
PA-L3892	TGT TTT TCC ATC TTG CCG TAA
2121R	TTA TCA GCG GAA GTA GCA AAT GTA
3892F	TTA CGG CAA GAT GGA AAA ACA
2557R	AGC CGT GCT CCA TTT TTC AGG
3318R	TGC GGT AAC ACT BTCA CTC CAG
2560F	GAA AAA TGG AGC ACG GCT TCT
2924F	CTT GGG CTG AAA CAA TGG GTT

^a^Primers used for amplification of the *pagA* gene. All other primers were used for sequencing.

Sequencing was performed on an Applied Biosystems 3100 genetic analyzer (Applied Biosystems) using BigDye terminator cycle sequencing ready reaction mix according to manufacturers instructions (Applied Biosystems). All sequence data were analyzed with the Lasergene 99 (DNASTAR, Madison, WI) software, which comprises several different programs: DNA sequences were assembled using the SeqMan program, and MegAlign was used to do sequence comparisons.

## Results

By MLVA, all 135 outbreak-related *B. anthracis* isolates had the following loci sizes: *vrrA* = 313, *vrrB*_1_ = 229, *vrrB*_2_ = 153, *vrrC*_1_ = 583, *vrrC*_2_ = 532, CG3 = 158, pXO1 = 123, and pXO2 = 141, resulting in genotype 62, as described by Keim et al. (6). In addition, the entire 2,294-bp *pagA* gene was sequenced from a subset of 42 isolates: including ten patient isolates, all four powder isolates, and 28 select environmental isolates. All isolates had an indistinguishable sequence, PA genotype I (Table 2).

**Table 2:** MLVA and *pagA* genotyping of *Bacillus anthracis* isolates^a,b^

*B. anthracis* strain	No. strains	*vrrA*	*vrrB*_1_	*vrrB*_2_	*vrrC*_1_	*vrrC*_2_	CG3	pXO1	pXO2	MLVA type	PA genotype
Outbreak-associated	317	313	229	153	583	532	158	123	141	62	I
Ames	1	313	229	153	583	532	158	123	141	62	I
NH (2000032764)	1	301	229	153	538	604	158	132	139	78	VI
NH (2000032760)	1	313	229	153	538	604	158	123	139	New^e^	VI
RI (2000032763)	1	313	229	162	538	604	158	132	139	71	VI
RI (2000032761)	1	313	229	162	538	604	158	132	139	71	VI
MA (2000032762)	1	313	229	153	538	604	158	132	143	New^e^	VI
State A (2002017388)	1	313	229	162	613	604	153	–	137	Pasteur^c^	NA^d^
Pasteur (LRN)	1	313	229	162	613	604	153	–	137	Pasteur^c^	NA^d^
Country B (2002007581)	1	313	229	162	613	532	158	129	141	45	I
Country B (2002007648)	1	313	229	162	613	532	158	129	141	45	I
Country B (2002007649)	1	313	229	162	613	604	153	–	137	Pasteur^c^	NA^d^
Country B (2002007650)	1	313	229	162	583	532	158	129	_	Sterne^c^	I
Country B (2002007651)	1	313	229	162	583	532	158	129	_	Sterne^c^	I

^a^MLVA, multiple-locus variable-number tandem repeat analysis; *pagA*, protective antigen gene; PA, protective antigen; –, loci not detected; NA, not applicable. ^b^Allele size for each VNTR locus is shown in addition to the MLVA and PA genotypes. ^c^No MLVA genotype assigned due to the lack of one of the virulence plasmids (pXO1 or pXO2). ^d^*pagA* not present in pXO1-cured strains and thus could not be assigned a PA genotype. ^e^New combination of alleles resulting in a new genotype. Genotype no. to be assigned at a later date.

Before *B. anthracis* was detected in the mail, we subtyped several isolates from cutaneous anthrax cases that occurred in the 1960s and 1970s in the eastern United States to determine if any were similar to the outbreak strain. Two isolates from Rhode Island were MLVA genotype 71, one New Hampshire isolate was genotype 78, while an additional New Hampshire isolate and a Massachusetts isolate each had unique combinations of alleles resulting in new genotypes**.** The *pagA* sequence of all five New England isolates was identified as PA genotype VI (Table 2)**.**

State A reported isolating *B. anthracis* (2002017388) from an envelope. This state was not in the vicinity of the 2001 outbreak. By MLVA, the isolate was shown to have been cured of pXO1 and had the same genotype as the Pasteur strain, used in laboratories as a control strain for various tests (Table 2).

Country B sent an isolate (2002007581) that was reportedly isolated from a letter to a private physician. MLVA identified the strain as genotype 45, which clearly distinguished it from the strain associated with the ongoing outbreak in the United States. In addition, four other isolates from the same facility were assayed by MLVA (2002007648–51), resulting in the identification of two Sterne strains, one Pasteur strain, and one additional strain of genotype 45 (Table 2).

MLVA and *pagA* sequencing were performed on clinical specimens collected from seven patients with laboratory-confirmed inhalational anthrax during the 2001 bioterrorism-associated anthrax outbreak. These methods have an unproven utility on clinical specimens, and further testing will be necessary for full evaluation. A total of 28 clinical specimens were analyzed by using MLVA, including: blood, CSF, pleural fluid, serum, lung tissue, and lymph node tissue (Table 3). All eight loci were detected in three specimens (two pleural fluids and one lymph node from patient 10). Of the eight loci examined, v*rrA* was detected in all nine specimens in which any of the MLVA loci were detected and on two occasions was the only locus detected. The *pagA* gene was successfully amplified and sequenced from 5 of 22 specimens analyzed (Table 3).

**Table 3:** Molecular subtyping by MLVA and *pagA* sequencing performed on 28 clinical specimens from seven patients with inhalational anthrax^a,b^

Patient no.^b^	Specimen type^c^	Interval after antimicrobial therapy (days)^d^	MLVA loci detected	*pagA*	*Bacillus anthracis* LRN PCR^e^
1	Pleural fluid	4	All negative	Negative	Positive
	Pleural fluid	4	All negative	Negative	Positive
	Blood	4	All negative	Negative	Negative
	Lung	4	All negative	ND	ND
	Lung	4	*vrrA*	ND	Negative
	Heart blood	4	*vrrA*, *vrrB_1_*	Negative	Negative
	Pericardial blood	4	All negative	Negative	Positive
2	Thoracentesis fluid	4	v*rrA*	Negative	Positive
	Serum	10	All negative	Negative	Positive
	Respiratory wash	4	All negative	Negative	Positive
	Pleural fluid	4	All negative	Negative	Positive
3	Blood culture	0	All negative	Negative	Positive
5	Blood culture	0	All negative	Negative	Positive
	Blood culture	0	All negative	Negative	Positive
6	Blood culture	0	*vrrA, vrrB_1_, vrrB_2_,,vrrC_2_*	Negative	Positive
10	Pleural fluid	1	*vrrA*, *vrrB_1_, vrrB_2_*, *vrrC_1_, vrrC_2_*, CG3, pXO1, pXO2	Positive	Positive
	Pleural fluid	1	*vrrA*, *vrrB_1_, vrrB_2_*, *vrrC_1_, vrrC_2_*, CG3, pXO1, pXO2	Positive	Positive
	Blood	1	All negative	Negative	Positive
	CSF	3	All negative	Negative	Positive
	Lung	3	*vrrA*, CG3	Negative	Positive
	Lymph node	3	*vrrA*, *vrrB_1_, vrrB_2_*, *vrrC_1_, vrrC_2_*, CG3, pXO1, pXO2	Positive	Positive
11	Pleural fluid	2	All negative	Positive	Positive
	Blood	2	All negative	Negative	Negative
	Blood culture	-1	All negative	ND	ND
	Blood culture	-1	All negative	ND	Positive
	Blood culture	-1	All negative	ND	ND
	Blood culture	-1	All negative	ND	Positive
	Lymph node	4	*vrrA*, *vrrB_1_, vrrB_2_*, *vrrC_1_, vrrC_2,_* CG3	Positive	Positive

^a^MLVA, multiple-locus variable-number tandem repeat analysis; *pagA*, protective antigen gene; LRN, Laboratory Response Network; PCR, polymerase chain reaction. ^b^Patients 1–10 described in Jernigan et al. (19) and patient 11 in Barakat et al (20) ^c^Specimens collected postmortem.  ^d^Number of days the specimen was collected following or before the initiation of antimicrobial therapy. Specimens collected the same day as the initiation of therapy were designated as day 0 but were collected before antibiotic therapy. ^e^Results using the Laboratory Response Network PCR assay for detection of *B. anthracis* during the outbreak (18).

## Discussion

During the 2001 anthrax investigation, molecular subtyping of *B. anthracis* by MLVA and *pagA* sequencing was important in linking cases to each other and to contaminated sites and in distinguishing isolates that were not related to this event. We used two methods for the molecular subtyping of *B. anthracis*: *pagA* sequencing and MLVA. All outbreak-associated isolates were identified as MLVA genotype 62 and PA genotype I. To date, MLVA genotype 62 has only been associated with a few isolates from herbivores in Texas and has not been identified in any *B. anthracis* strains originating in eastern United States or anywhere else in the world. None of the New England isolates analyzed in this study were MLVA genotype 62 or PA genotype I. All five were of PA genotype VI, while MLVA identified two isolates as genotype 71, one as genotype 78, and two as new genotypes. Genotype 62 is also the genotype of the Ames strain commonly used in research laboratories worldwide and frequently used in animal challenge studies (11–16). The sequence of *pagA* from the outbreak strain, PA genotype I, was also identical to that of the Ames strain; thus, the outbreak *B. anthracis* strain is indistinguishable from the Ames strain based on the examination of the eight MLVA loci and the *pagA* sequence. Recently, comparative genome sequencing detected only four differences between the chromosomes of the outbreak strain (Florida isolate) and Ames (Porton) isolate (17).

Molecular subtyping of isolates immediately upon their arrival to the laboratory allowed for instant confirmation that the cases were caused by the same strain and thus for linking cases to environmental contamination and to the powder-containing envelopes. The speed of the MLVA allowed for genotype identification within 8 hours of receiving the isolates. In addition to linking the cases, molecular subtyping was invaluable in determining if *B. anthracis,* isolated from around the world during the same time period, were potentially related to the ongoing outbreak in the United States. The level of discrimination provided by MLVA, allowed for non-outbreak isolates to be rapidly and easily distinguished.

While both MLVA and *pagA* sequencing are primarily used for molecular subtyping of isolates, we were also able to amplify the eight MLVA loci and *pagA* directly from a limited number of available clinical specimens. Although this event was not a prospective case-control study, amplification was most successful from pleural fluid and lymph node specimens. Similar results were demonstrated with a *B. anthracis* specific real-time PCR assay (18). Amplification of the MLVA loci and *pagA* was not very successful from blood cultures even when taken before antibiotic therapy. The lack of success with blood cultures was not because of a complete inhibition of PCR since the *B. anthracis* LRN PCR assay was positive on these specimens. Of the MLVA loci, *vrrA* was the most readily amplified (9 of 28), probably because *vrrA* amplification is more sensitive compared to the other MLVA loci. However, limits of detection for each of the loci has not been evaluated.

For a single patient (patient 10), we were able to amplify all eight MLVA loci and determine the genotype of the *B. anthracis* strain without having the isolate itself. In this instance, MLVA was used directly on DNA extracted from pleural fluid and genotype 62 was identified. *B. anthracis* was not successfully cultured from that same pleural fluid sample. The *pagA* gene was amplified and sequenced from the same DNA specimen and identified as PA genotype I. MLVA and *pagA* amplification were attempted on DNA extracted from blood drawn from this patient the same day as the pleural fluid but failed to detect any of the loci, suggesting more efficient clearance of the bacilli from the blood or less sensitivity of these molecular approaches on blood compared to pleural fluid. Again, the negative result on blood was not because of complete inhibition of PCR since the *B. anthracis* LRN PCR assay on this sample was positive. At a later date, when the Centers for Disease Control and Prevention received the isolate originally cultured from this patient at the local medical facility where the patient was treated, the isolate was confirmed to be genotype 62. Despite the fact that *B. anthracis* was not successfully cultured from any of these clinical specimens taken after the initiation of antimicrobial therapy, we were able to amplify the MLVA loci and *pagA* from some of these specimens.

The entire chromosomal sequence of the *B. anthracis* Ames strain (available from: URL: www.tigr.org) is now available and has been compared to the chromosomal sequence of the outbreak (Florida) isolate (17). While sequencing and comparing *B. anthracis* genomes are not likely to be useful for rapidly identifying isolates during an outbreak investigation, the data generated from such comparisons may identify new loci, which could be targets for methods such as MLVA and can be done rapidly on large numbers of isolates from patients, the environment, and on DNA from clinical specimens.
